# Kaolin-derived zeolite enables high-performance carbon capture with gigaton-scale potential

**DOI:** 10.1093/nsr/nwag064

**Published:** 2026-04-13

**Authors:** Jinlei Li, Junyan Li, Siyuan Fang, Ge Zhang, Pu Zhang, Sonia Shum, Zimo Zhang, Yi Cui

**Affiliations:** Department of Materials Science and Engineering, Stanford University, Stanford, CA 94305, USA; Department of Materials Science and Engineering, Stanford University, Stanford, CA 94305, USA; Department of Materials Science and Engineering, Stanford University, Stanford, CA 94305, USA; Department of Materials Science and Engineering, Stanford University, Stanford, CA 94305, USA; Department of Materials Science and Engineering, Stanford University, Stanford, CA 94305, USA; Department of Materials Science and Engineering, Stanford University, Stanford, CA 94305, USA; Department of Materials Science and Engineering, Stanford University, Stanford, CA 94305, USA; Department of Materials Science and Engineering, Stanford University, Stanford, CA 94305, USA; Stanford Institute for Materials and Energy Sciences, SLAC National Accelerator Laboratory, Menlo Park, CA 94025, USA; Department of Energy Science and Engineering, Stanford University, Stanford, CA 94305, USA

**Keywords:** CO_2_ capture, scalable adsorbent synthesis, passive thermal cycling

## Abstract

Gigaton-scale carbon dioxide (CO_2_) capture is an indispensable part of the way towards global carbon neutrality, but has lagged in developing an adsorbent that simultaneously has high performance, low cost, and scalability from earth-abundant raw materials coupled with industrially compatible synthesis processes. Here we discover that high-performing CO_2_ adsorbent of Linde Type A (LTA) zeolite can be converted from ubiquitous kaolin clay (reserves >30 gigatons) via scalable processes and exhibits record high CO_2_ uptake with good cycling stability. The synthesis route, comprising mainly calcination and a hyperthermal reaction, is readily compatible with existing industrial infrastructure and avoids the use of complex or toxic chemicals. Benefiting from an optimized crystal structure for CO_2_ trapping, the material achieves CO_2_ adsorption capacities that surpass all previously reported clay-derived zeolites across a wide concentration range, from ambient air (∼400 ppm) to flue gas conditions (<20%). It also maintains stable performance over 50 adsorption–desorption cycles. Beyond material and method development, we provide a proof-of-concept showing that integrating radiative cooling for CO_2_ adsorption and solar heating for sorbent regeneration could enable a low-carbon pathway for passive sorbent operation. This study offers a feasible route to explore scalable carbon capture using widely available materials and passive energy strategies.

## Teaser

This work reports a scalable and sustainable carbon capture approach based on earth-abundant resources and renewable energy inputs.

## INTRODUCTION

Large-scale carbon dioxide (CO_2_) capture is widely recognized as a critical technology for mitigating climate change and a cornerstone of global sustainable economic development [1–3]. Among the various capture strategies, solid adsorbents have attracted growing attention due to their lower energy requirements and operational simplicity, offering key advantages over traditional solvent-based systems [4–6]. In response to the pressing need for effective CO_2_ mitigation, research efforts have increasingly focused on the design of advanced adsorbent materials, resulting in notable progress over the past few decades [7–18].

Nevertheless, developing an adsorbent that simultaneously offers high performance, low cost, and scalability—all derived from earth-abundant raw materials and through industrially compatible synthesis processes—remains a major challenge and is still a central focus of this field. To achieve capture at the gigaton scale, materials abundant enough to meet such enormous demands must be identified. Naturally occurring clays, ubiquitously distributed across the planet, stand out as one of the particularly promising candidates.

In this work, we demonstrate that Linde Type A (LTA) zeolite, a high-performing CO_2_ adsorbent, can be synthesized directly from widely available kaolin clay (reserves >30 gigatons) using scalable, industrially compatible processes. The resulting LTA zeolite possesses a crystal structure optimized for CO_2_ accommodation and fixation, enabling a record-high CO_2_ uptake across a wide concentration range (400 ppm to 20%), surpassing all previously reported clay-derived materials. The material also exhibits excellent cycling stability over 50 adsorption–desorption cycles without signs of degradation. Furthermore, we provide a proof-of-concept that the adsorption–desorption process could be driven by passive approaches, using radiative cooling to enhance adsorption and solar heating for regeneration. Together, these findings point to a practical and scalable approach for sustainable CO_2_ capture based on earth-abundant materials and renewable energy inputs.

## RESULTS

### Concept illustration

**Figure 1.**
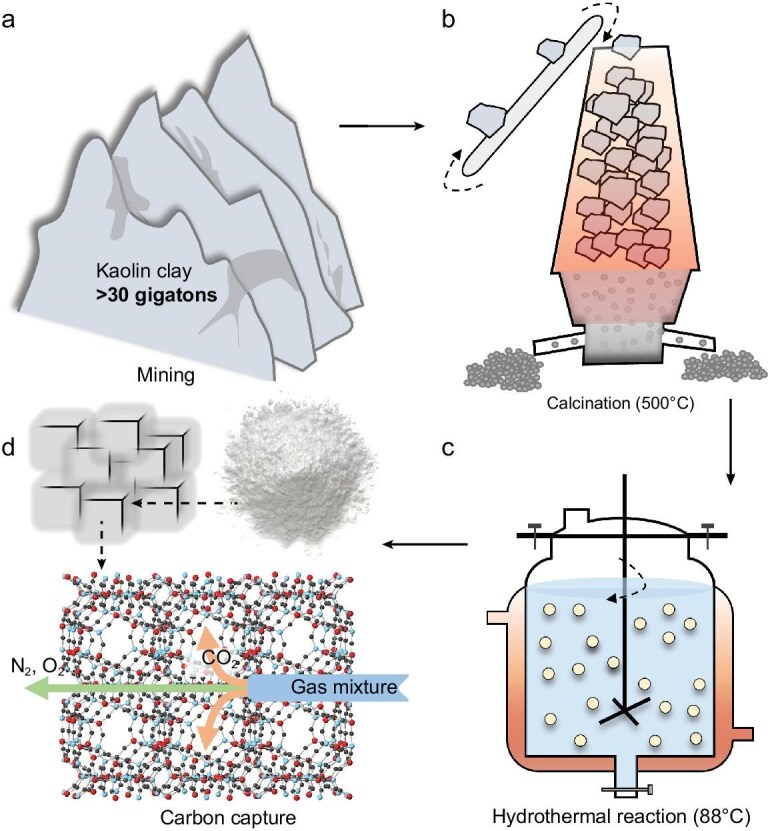
Synthesis of LTA zeolite from earth-abundant kaolin clay for efficient CO_2_ capture. (a) Kaolin clay, with proven reserves exceeding 30 gigatons, is a promising material for scalable CO_2_ adsorbent production. (b and c) Leveraging industrially compatible methods of calcination and hydrothermal processes, kaolin clay is converted into LTA zeolite. (d) The optimized crystal structure of the LTA zeolite enhances CO_2_ trapping, allowing selective separation and capture of CO_2_ from mixed gases, significantly outperforming the original kaolin clay.

Figure 1 illustrates the concept of synthesizing LTA zeolite from earth-abundant kaolin clay to achieve high-performance CO_2_ capture. Kaolin clay is widely distributed across all continents, with total proven reserves exceeding 30 gigatons (Fig. 1a) [19,20]. We aim to transform kaolin clay into LTA zeolite, which has good CO_2_ adsorption capacity over a wide concentration range. Our laboratory trials demonstrate that by employing two sequential and industrially compatible processes, calcination at 500°C followed by hydrothermal synthesis at 88°C (Fig. 1b and c), LTA zeolite can be successfully produced from raw kaolin without the need for complex or novel chemical additives (see Methods for further details). The resulting LTA zeolite exhibits a crystal structure with strong affinity for CO_2_, while maintaining significantly lower interactions with other gases such as nitrogen, thereby enabling effective and selective CO_2_ capture (Fig. 1d).

### Structural and morphological characterizations

**Figure 2.**
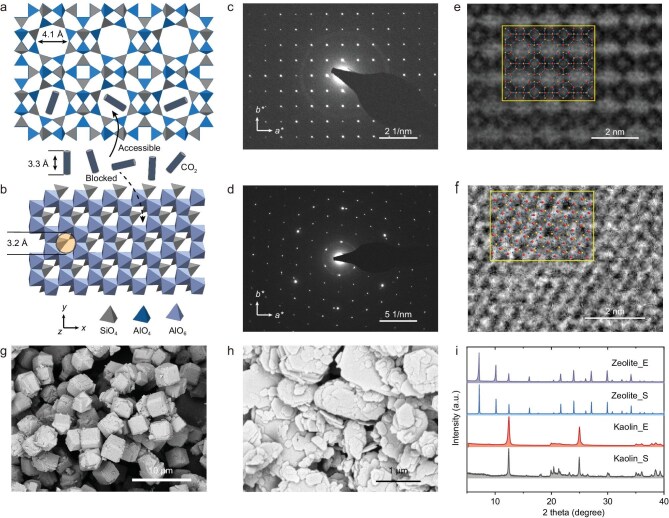
Material structural and morphological characterizations. (a and b) Crystal structures of LTA zeolite and kaolin clay, respectively. The LTA zeolite’s crystal structure allows for CO_2_ molecules to enter and be trapped on active sites, whereas the void space in kaolin clay is insufficient for CO_2_ accommodation. (c and d) SAED patterns of the as-synthesized LTA zeolite and kaolin clay, respectively. (e and f) HRTEM images of the LTA zeolite and kaolin clay, respectively. Insets show schematic representations of the crystal structures in a and b in a ball-and-stick form, confirming consistency with the theoretical structures. (g and h) SEM images of the LTA zeolite and kaolin clay, respectively. (i) XRD patterns of LTA zeolite and kaolin clay, with ‘E’ and ‘S’ denoting experimental and standard data, respectively.

The atomic structure of the synthesized LTA zeolite is fundamentally distinct from that of the parent kaolin clay, a transformation that is critical for enabling effective CO_2_ adsorption (Fig. 2a). The pore opening of the LTA crystal is 4.1 Å, which is larger than the kinetic diameter of CO_2_ molecules (3.3 Å), thereby allowing CO_2_ to readily diffuse into the internal void spaces for adsorption on active sites. In contrast, the interlayer spacing and the distance of the crystal face in kaolin are only 2.8 and 3.2 Å, respectively, smaller than the size of CO_2_ molecules, preventing access to the internal surfaces and thereby limiting its gas adsorption capability (Fig. 2b).

Selected area electron diffraction (SAED) patterns validate the structural transformation. The SAED pattern of the LTA zeolite (Fig. 2c) displays a series of sharp and regularly spaced diffraction spots, consistent with a well-ordered cubic crystal structure, different from that of kaolin clay (Fig. 2d). High-resolution transmission electron microscopy (HRTEM) images provide additional atomic-scale insights: Fig. 2e reveals a periodic lattice corresponding to the LTA framework structure outlined in Fig. 2a, while Fig. 2f shows a near-hexagonal structure typical of kaolin. These HRTEM observations confirm the successful reorganization of the atomic structure during synthesis.

At a larger scale, scanning electron microscopy (SEM) was employed to study the morphology of the samples. The SEM images reveal that the as-synthesized LTA zeolite predominantly forms cubic microparticles with an average size of ∼5 μm (Fig. 2g), which is in stark contrast to the lamellar structure observed in the raw kaolin clay (Fig. 2h and Fig. S1). Powder X-ray diffraction (XRD) patterns provide further confirmation of phase purity and crystallinity. As shown in Fig. 2i, the XRD peaks of the synthesized LTA zeolite closely match the standard diffraction pattern of pure LTA, with no detectable peaks corresponding to kaolin, indicating a complete phase transformation. Moreover, the sharp and intense diffraction peaks reflect the high crystallinity of the LTA zeolite, a desirable feature that enhances its adsorption performance and stability of CO_2_ capture.

### Material adsorption characterizations

**Figure 3.**
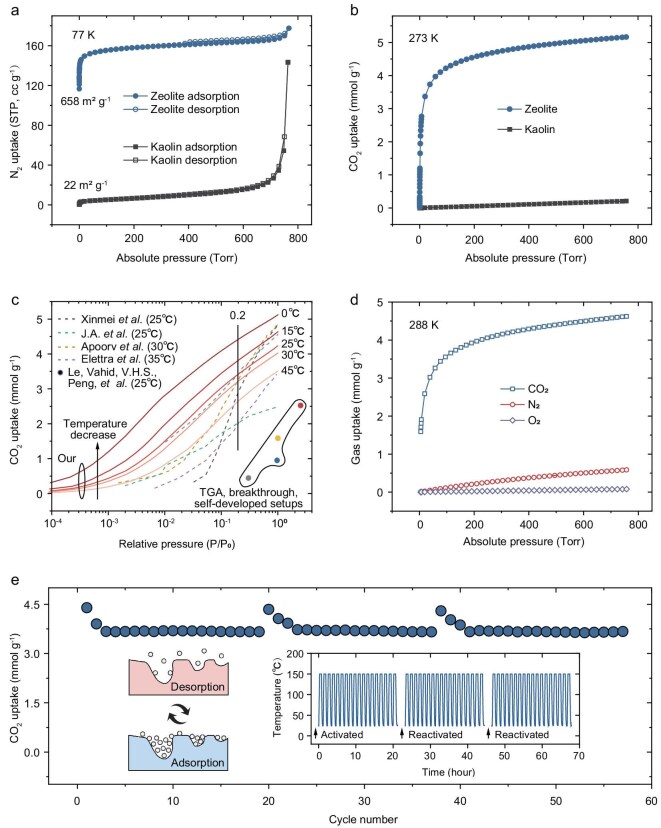
Material adsorption characterizations. (a) N_2_ sorption isotherms at 77 K. The as-prepared LTA zeolite exhibits a significantly higher specific surface area compared to its precursor, kaolin clay, due to its distinct crystal structure. (b) CO_2_ sorption isotherms at 273 K. LTA zeolite shows superior CO_2_ adsorption performance over kaolin clay, attributed to its well-defined crystal structure and high crystal quality. (c) Performance comparison with previously reported clay-sourced zeolites (see references in the main text). Lines represent isotherm test results, while dots correspond to test results by TGA, breakthrough, and self-developed setups. The LTA zeolite demonstrates the best CO_2_ capture performance (when CO_2_ concentration is <20%), with potential for further improvement via cooling, as demonstrated later. (d) Sorption isotherms of CO_2_, N_2_, and O_2_ on LTA zeolite at 288 K, indicating strong CO_2_ affinity and high CO_2_/N_2_ and CO_2_/O_2_ selectivity. (e) Adsorption–desorption cycling stability test via TGA. The inset illustrates the experimental method, with adsorption at 25°C (for pure CO_2_) and desorption at 150°C. The spare time in the temperature-time curve denotes complete reactivation for the sample at 450°C for 30 minutes. The LTA zeolite exhibits good cycling stability, with no signs of degradation.

The crystal structure of the synthesized LTA zeolite is more favorable than that of kaolin clay for CO_2_ capture. Nitrogen (N_2_) adsorption measurements at 77 K confirm that the LTA zeolite possesses a specific surface area of up to 658 m² g^-1^, which is nearly 30 times greater than that of kaolin, which exhibits a modest surface area of only 22 m² g⁻¹ (Fig. 3a). This dramatic increase in surface area, combined with the optimized pore size, endows the LTA zeolite with excellent CO_2_ uptake performance. Specifically, under a CO_2_ pressure of ∼760 Torr at 273 K, the LTA zeolite achieves a CO_2_ adsorption capacity of 5.2 mmol g⁻¹ (Fig. 3b), nearly 25 times higher than that of the raw kaolin clay, which reaches only 0.2 mmol g⁻¹. This stark contrast highlights the crucial role of the atomic-scale structural reorganization from kaolin to LTA zeolite in dramatically enhancing CO_2_ capture capability.

We further benchmarked the performance of our LTA zeolite against previously reported clay-derived zeolites and commercial high-performance 13X zeolite. As shown in Fig. 3c and Fig. S2, our sample exhibits the highest CO_2_ uptake across a wide range of CO_2_ concentrations, from 400 ppm (typical of ambient air) to 20% (representative of flue gas), demonstrating its versatility for both direct air capture (DAC) and post-combustion capture scenarios [21–29]. Additionally, lowering the adsorption temperature further amplifies the performance advantage of our sample, an effect that will be discussed in more detail in the following sections.

Beyond uptake capacity, the selectivity and cycling stability of the LTA zeolite were also assessed. As shown by the adsorption isotherms at 288 K in Fig. 3d, the LTA zeolite exhibits a much stronger affinity for CO_2_ than for N_2_ and O_2_, resulting in excellent CO_2_/N_2_ and CO_2_/O_2_ selectivity (Fig. S3). To evaluate stability, the LTA zeolite was subjected to over 50 adsorption–desorption cycles using thermogravimetric analysis (TGA), with adsorption carried out at 25°C and desorption at 150°C (Fig. 3e, see more experimental details in the Methods section). The CO_2_ uptake remained highly stable after the initial 2–3 cycles, confirming the material’s excellent reusability. It is also worth noting that the three groups of regeneration tests show high similarity, suggesting the sample is capable of undergoing more same operations (regeneration cycles). However, we observed that after ∼20 cycles, full regeneration at higher temperatures becomes necessary; otherwise, the capacity decreases rapidly. This behavior likely arises from gradual accumulation of residual CO_2_ species that cannot be completely removed at 150°C, though the exact decay mechanism remains unclear. Further characterization (e.g. *in-situ* IR and solid-state NMR) will be required to identify these strongly bound species and guide future material-level strategies in order to lower the regeneration energy. From a process-engineering standpoint, the use of low-carbon heat sources, for example solar thermal energy, geothermal heat, and industrial waste heat, offers a route to reduce the operational carbon footprint and reliance on fossil-derived heating. Additionally, low-temperature vacuum swing or assisted regeneration approaches may also be helpful to lower the regeneration temperature.

### Enhanced CO_2_ capture and regeneration by thermal management

**Figure 4.**
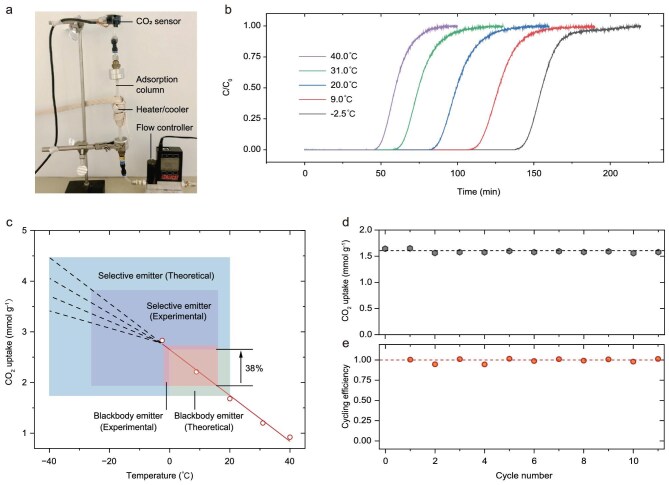
Enhanced CO_2_ capture and regeneration by thermal management. (a) Experimental setup. The system includes a flow controller for regulating gas flow rate, a heater/cooler for controlling column temperature, an adsorption column for loading samples, and a CO_2_ sensor for measuring outlet CO_2_ concentration; 1% CO_2_ was used in this test. (b) Breakthrough results at different column temperatures. Cooling effectively enhances CO_2_ uptake performance. (c) Linear fit of CO_2_ uptake and column temperature (red circles). Further temperature reduction continues to increase uptake, though the trend becomes uncertain (indicated by the dashed lines). The left edges of the shadowed areas show temperature decreases enabled by various radiative cooling scenarios from the right edges. (d) Cycling tests by temperature swing. Repeated adsorption (at 20°C) and desorption (at 200°C) cycles. (e) Cycling efficiency (from d). Defined by the quotient of CO_2_ uptake at cycle *n* + 1 divided by CO_2_ uptake at cycle *n*.

As shown in Fig. 3c, the CO_2_ adsorption process exhibits a strong temperature dependence. To further utilize this behavior, we employed a breakthrough setup to systematically study the impact of temperature on the adsorption characteristics of the as-prepared LTA zeolite (Fig. 4a, using 1% CO_2_; see Methods for details). The breakthrough curves in Fig. 4b, collected at temperatures ranging from –2.5 to 40°C, clearly reveal that lower operating temperatures significantly enhance the CO_2_ adsorption capacity of the LTA zeolite. The corresponding CO_2_ uptake values, summarized in Fig. 4c (red circles), demonstrate a nearly linear increase with decreasing column temperature, suggesting the feasibility of leveraging cooling strategies to further improve adsorption performance. In practice, such cooling can be achieved through geocooling, seasonal/diurnal energy storage, or radiative cooling (a fully passive process that enables cooling without the need for external energy input from fossil fuel consumption, see previous works for more details of this technique [30–32]). To exemplify this potential, we here show four representative radiative cooling scenarios as shaded regions in Fig. 4c. Even in the simplest case, where experimental studies have demonstrated radiative cooling from 15°C down to 0°C, the CO_2_ uptake increases by ∼38% compared to ambient conditions. More aggressive radiative cooling strategies in the other three cases are expected to be capable of achieving even greater performance enhancements.

Using the same breakthrough setup, we also evaluated the desorption/regeneration behavior and cycling of the LTA zeolite. As shown in Fig. S4, the sample can be effectively regenerated at 160°C and maintains stable CO_2_ uptake across more than 10 adsorption–desorption cycles, which are consistent with the TGA results presented in Fig. 3e. Notably, when the regeneration temperature is increased to 200°C, the LTA zeolite exhibits a CO_2_ uptake capacity identical to that achieved after the initial activation at 300°C (Fig. 4d). This finding indicates that heating to ≥200°C enables near-complete regeneration of the material (Fig. 4e). Importantly, such regeneration temperatures are practically achievable using waste heat generated in the process chain of energy conversion from primary energy [33,34] or utilizing solar heating, another passive, fossil-free solution. Prior studies have indeed shown that solar-driven heating to ≥200°C can be realized with planar solar absorbers even without the need for optical concentrators [35].

### A low-carbon route for adsorbent cycling operation

**Figure 5.**
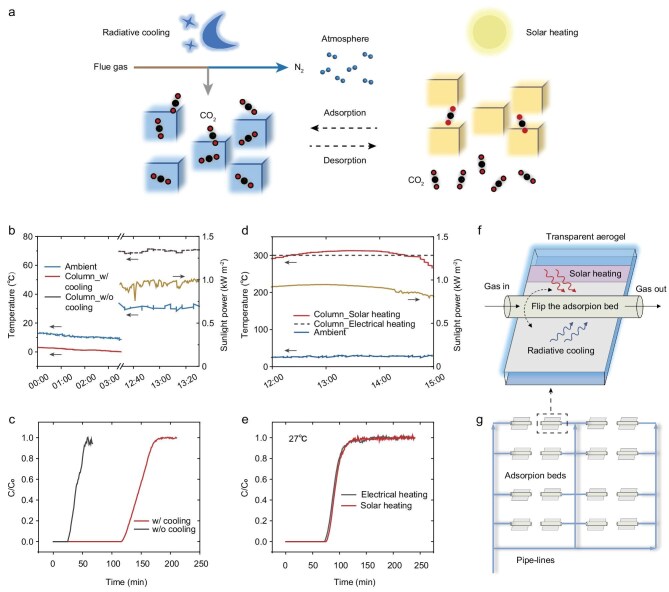
Passive CO_2_ capture–release cycle. (a) A carbon-free route for adsorbent operation is proposed by integrating radiative cooling for adsorption enhancement with solar heating for regeneration. (b) Temperature (blue, red, black lines) and sunlight power (yellow line) records. (c) Breakthrough curves for LTA zeolite with and without cooling. CO_2_ uptake is significantly improved by the cooling effects, which include exploiting radiative cooling, utilizing the day/night temperature difference, and avoiding sunlight heating. (d) Records of temperature profiles (blue and red lines) and sunlight power (yellow). Regeneration of the LTA zeolite was compared between solar heating using an evacuated tube and electrical heating. (e) Breakthrough results. Solar heating demonstrates regeneration efficiency on par with electrical heating. (f and g) Conceptual illustration of a passive CO_2_ capture–release farm. The cycle was operated by flipping the adsorption bed. Heat and cold energy are delivered to the column via the wings.

As discussed above, the LTA zeolite can be regenerated via a heating process, and its adsorption performance can be enhanced via lowering the adsorption temperature. We here provide a proof-of-concept demonstration for a low-carbon route for adsorbent cycling operation by integrating radiative cooling for adsorption enhancement and solar heating for desorption (Fig. 5a). As demonstrated in Fig. 5b, radiative cooling enables a temperature reduction of ∼10°C during nighttime field tests (see Figs S5 and S6 for more experimental details). The column (fabricated from stainless steel) reaches daytime temperatures that are ∼70°C higher than nighttime temperatures under radiative cooling. This substantial thermal contrast significantly affects the CO_2_ adsorption behavior, as evidenced by the breakthrough curves shown in Fig. 5c. Specifically, CO_2_ adsorption during nighttime with radiative cooling is more than three times greater than that during the daytime, highlighting the substantial performance gains achieved through passive cooling strategies.

Following adsorption, we employed solar heating to regenerate the LTA zeolite. Thanks to effective suppression of thermal losses via convection and radiation control, the solar heater was able to achieve temperatures near 300°C under field conditions (Fig. 5d, see Figs S7 and S8 for the experimental setup). We also subjected the LTA samples to electrical heating at 300°C for 3 hours. Remarkably, the regeneration performance achieved by solar heating matches that of conventional electrical heating, as shown in Fig. 5e, confirming that effective regeneration can be accomplished entirely with carbon-free solar energy.

The above results collectively demonstrate that the combination of radiative cooling and solar heating offers a promising route for realizing sustainable, fossil-fuel-free CO_2_ capture and release cycles. We here propose a conceptual Janus prototype device with opposite thermal functions, solar heating on one side and radiative cooling on the other, for integrated and enhanced CO_2_ capture–release cycles via temperature swing (Fig. 5f). Preliminary outdoor tests provide an indication of the feasibility of this idea (Fig. S9), though long-term performance under varied environmental conditions will require more careful evaluation in future studies. Looking ahead, such a concept could potentially be scaled into a CO_2_ capture–release farm, analogous to a photovoltaic installation, although this remains a future design vision (Fig. 5g). An energy and area balance analysis related to this concept is provided in Supplementary Note 1.

### First-pass techno-economic, energy consumption, and carbon footprint assessments

To assess the economic prospects of kaolin-derived zeolite for CO_2_ capture driven by solar heating and radiative cooling, we carried out a techno-economic analysis for flue gas capture (Supplementary Note 2 and Fig. S10). The results show that the capture cost per ton of CO_2_ declines sharply as the number of adsorption–desorption cycles increases (Fig. S11). Notably, once the system operates beyond ∼50 cycles, the projected cost drops below that of conventional CO_2_ capture technologies commonly used across major industrial sectors. The preliminary results indicate that the combination of low-cost raw materials and passive thermal management could enable a competitive cost trajectory for future CO_2_ capture applications.

Beyond economic cost, evaluating the operational energy penalty is also essential. The intrinsic CO_2_ adsorption heat provides a direct indicator of the regeneration energy demand. We measured the isothermal adsorption curves of the as-prepared zeolite at 273, 298, and 318 K (Fig. S12a) and calculated the adsorption heat. As shown in Fig. S12b, the adsorption heat at low CO_2_ coverage is ∼50 kJ mol⁻¹. With increasing CO_2_ loading, the adsorption heat is expected to decrease due to the progressive occupation of weaker adsorption sites and micropore filling. The adsorption heat is lower than that typically reported for amine-based adsorbents (60–100 kJ mol⁻¹ [36]). Using the same regeneration protocol as in the TGA cycling tests, we further estimate the energy consumption for releasing 1 ton of CO_2_ from the zeolite via solar heating (Supplementary Note 3). This requires ∼60 kWh of heat. In comparison, conventional temperature-swing adsorption systems typically require a minimum of ∼500 kWh per ton of CO_2_ [37–39]. Although our estimation is based on a simplified model and may not capture all process-level losses, the large margin suggests substantial headroom for system optimization and highlights the strong potential of the adsorbent fabrication and desorption strategy. For the adsorption step, radiative cooling can further enhance CO_2_ uptake. In conventional industrial systems, active cooling is generally not implemented because the electricity consumption often outweighs the gain in adsorption. In contrast, radiative cooling offers a fully passive pathway. Although a direct energy-consumption comparison is not possible due to the lack of industrial analogues, our analysis shows that the cumulative cooling energy harvested by a radiative cooler over its lifetime exceeds both the economic cost and carbon emissions associated with its fabrication. This indicates that radiative cooling can function as a cost-effective and carbon-negative auxiliary process for enhancing CO_2_ adsorption efficiency.

Finally, assessing the carbon footprint of the material and method reported here provides insight into their overall carbon capture performance. In this estimation, CO_2_ adsorption by the zeolite contributes a negative carbon footprint, while emissions arise primarily from adsorbent synthesis and operational energy (Supplementary Note 4). We calculated the net carbon footprint by combining captured and emitted CO_2_ and plotted it as a function of adsorption–desorption cycles (Fig. S13). The results show that the footprint becomes negative after just four cycles, indicating that the initial emissions from synthesis are fully offset by the zeolite’s CO_2_ capture. With continued cycling, the material enables substantial net carbon removal as cumulative capture increases, demonstrating the potential of this approach for effective carbon mitigation.

## CONCLUSION AND DISCUSSION

In summary, this work demonstrates that high-performing LTA zeolite can be produced from earth-abundant kaolin clay through scalable processes, and provides a proof-of-concept for passive CO_2_ adsorption driven by radiative cooling and solar regeneration. We discover that the conversion of earth-abundant kaolin clay (with reserves of more than 30 gigatons) into LTA zeolite offers a highly promising and scalable pathway toward high-performance CO_2_ capture at the gigaton scale. The synthesis process relies on industrially compatible techniques without the need for complex or hazardous chemicals, underscoring its practical feasibility for large-scale implementation. The resulting LTA zeolite achieves a record-high CO_2_ uptake, surpassing all previously reported clay-derived zeolites across a wide concentration range, from direct air capture (400 ppm) to flue gas capture (<20%). In addition, the material demonstrates excellent cycling stability, maintaining its adsorption performance over 50 adsorption–desorption cycles. Beyond material and method development, we exemplify a fully passive CO_2_ capture–release cycle by integrating radiative cooling for adsorption enhancement with solar heating for regeneration. Together, these advances pave a new avenue for a scalable and sustainable carbon capture solution based on earth-abundant resources and renewable energy inputs.

Despite these promising results, several aspects warrant further investigation to enable practical deployment. First, although water vapor lowers CO_2_ uptake by ∼10% under our test conditions (Figs S14 and S15), the effect is moderate. Moisture remains a non-negligible competitive adsorbate for zeolites, and future studies may investigate mitigation strategies including hydrophobic modification or system-level moisture control. Second, the long-term stability of the zeolite under repeated thermal cycling (hundreds of cycles) and exposure to environmental contaminants (e.g. SO_x_, NO_x_) requires further evaluation. Third, while the passive CO_2_ capture–release cycle shows strong potential, its performance under variable real-world climates and prolonged outdoor operation remains to be fully analyzed. Finally, the cost and environmental impacts of large-scale kaolin-to-zeolite conversion and passive CO_2_ capture operations should be quantitatively assessed through more comprehensive techno-economic and life-cycle analysis. While substantial challenges remain, this work provides a promising starting point for scalable, sustainable carbon capture using earth-abundant materials and renewable energy. We anticipate that continued collective efforts will accelerate the advancement of this material/approach toward realizing the ambitious goal of gigaton-scale CO_2_ removal.

## MATERIALS AND METHODS

Materials and methods are available in the online Supplementary data.

## Supplementary Material

nwag064_Supplemental_File
