# Supplementary material for: Kaolin-derived zeolite enables high-performance carbon capture with gigaton-scale potential
Source: Natl Sci Rev. 2026 Apr 13;13(8):nwag064. doi: 10.1093/nsr/nwag064 (PMC13127140; doi:10.1093/nsr/nwag064)
Supplement: nwag064_Supplemental_File [file nwag064_supplemental_file.pdf]

Supplementary Data for

Kaolin-Derived Zeolite Enables High-Performance Carbon Capture with Gigaton-Scale Potential

Jinlei Li, Junyan Li, Siyuan Fang, Ge Zhang, Pu Zhang, Sonia Shum, Zimo Zhang, Yi Cui

Correspondence to: Yi Cui (yicui@stanford.edu)

This PDF file includes:

Materials and methods
Supplementary Notes
Supplementary Figures
Supplementary References

Materials and methods

Chemicals

All chemicals used in this study were purchased from Sigma-Aldrich and used without further purification.

Synthesis of LTA zeolite from kaolin clay

The kaolin clay was first calcined at 500 °C for 3 h under an air flow, with the calcination temperature determined via XRD analysis (Fig. S16). Subsequently, 1 g of the calcined kaolin was mixed with 5 mL of 3 mol L^{-1} sodium hydroxide solution and stirred at room temperature for 6 hours. The resulting mixture was then transferred to a 25 mL hydrothermal reactor and heated at 88 °C for 12 hours. The solid product was separated, thoroughly washed with deionized water, and dried at 100 °C overnight. Finally, 0.3 g of the as-prepared powder was mixed with 30 mL of 1 mol L^{-1} calcium nitrate solution and maintained at 80 °C for 24 hours. The solid product was again separated, washed, and dried. The soak treatment in calcium nitrate solution was done twice. Samples were collected for subsequent tests. Material and water balance analysis for the alkaline treatment and Ca-exchange processes indicates that most water, 42% of the sodium hydroxide, and 96% of the calcium nitrate can be recovered and reused for the next batch (Supplementary Note 5 and Figs. S17-19). In addition, both the alkaline treatment and Ca-exchange steps show good tolerance to impurities present in the raw kaolin (Supplementary Note 6).

Adsorption isotherms

All CO_2, N_2 and O_2 adsorption isotherm measurements were performed using an Autosorb iQ (Quantachrome/Anton Paar). Prior to testing, the LTA zeolite was activated at 340 °C for 6 hours, while the kaolin clay was degassed at 150 °C for 6 hours. A circulating water bath was employed for temperature control. For nitrogen adsorption measurements at 77 K, liquid nitrogen was used to maintain a stable temperature.

Material characterizations

SAED and HRTEM images were acquired using an FEI Titan Environmental TEM. SEM images were obtained with a Thermo Scientific Apreo S LoVac SEM. XRD patterns were collected using an Empyrean XRD (Cu Kα source, Malvern Panalytical). The elemental concentrations of kaolin and zeolite were measured via XRF Spectrometer (Spectro Xepos HE). TGA tests were performed using a Q5500 of TA Instruments. Air was used as the carrier gas if not specified.

TGA cycling test

Samples were first activated by heating to 450 °C under a 50 sccm helium flow for 30 minutes. After cooling to 25 °C, a 50 sccm flow of pure CO_2 was introduced for adsorption testing. During cycling, the sample was regenerated by heating to 150 °C under a 50 sccm helium flow. Complete reactivation at 450 °C for 30 minutes was carried out after around 20 adsorption–desorption cycles. Sample mass was continuously monitored and recorded for subsequent analysis.

Breakthrough test

A 0.5 g sample was loaded into a quartz tube with an outer/inner diameter of 12.68/7.79 mm and a length of 19.50 cm. The void space was filled with sand, 2 mm quartz balls, and deactivated glass wool, following the structure of a commercial indicating oxygen trap (Z-PURE, TRP-202237-B). During outgassing, the column was heated using a flexible heating tape under a 50 sccm helium flow. After naturally cooling to room temperature, the column was wrapped with plastic tubing connected to a circulating water system for temperature control. Once the column temperature stabilized, a 20 sccm flow of 1% CO_2 balanced with N_2 was introduced to initiate the adsorption process. CO_2 concentration at the outlet was continuously monitored and recorded using a CO_2 sensor (SprintIR-6S-5, CO_2 Meter) connected to a laptop. A control column without LTA zeolite was tested to establish a CO_2 adsorption baseline. All data presented in Fig. 4 were baseline-corrected accordingly. For the ambient-air breakthrough test, air was collected inside the Gordon & Betty Moore Materials Research Building at Stanford and pressurized

using a CRAFTSMAN 6-gallon portable air compressor before use. The air humidity was ~40% RH during collection.

Field tests of passive CO_2 capture and release

The column assembly and breakthrough tests followed procedures similar to those described above, with the glass tube replaced by a stainless steel tube with an outer/inner diameter of 9.56/7.56 mm and a length of 12 cm. For the cooling tests, the column was outgassed at 250 °C for 3 hours using a flexible heating tape under a 50 sccm N_2 flow. Nighttime and daytime adsorption tests were initiated at 12:00 a.m. and 12:30 p.m., respectively, using a 20 sccm flow of 1% CO_2 balanced with N_2. Column temperatures were left uncontrolled during these tests. In the solar heating tests, the column was inserted into an evacuated solar heating tube and then allowed to cool naturally to ambient temperature. During adsorption, the column temperature was maintained at 27 °C using a flexible heating tape. For electrical heating tests, the column was outgassed at 300 °C for 3 hours. During the multi-cycle breakthrough test, the sample was outgassed from 11:00 a.m. to 2:00 p.m. under a 50 sccm N_2 flow, followed by CO_2 adsorption starting at 12:00 a.m. using a 20 sccm flow of 1% CO_2. The column was oriented with the solar heating material facing the sky during the day and the radiative cooling material at night. To replicate the solar heating conditions achieved with transparent aerogel, electrical heating was applied as a supplement, enabling an outgassing temperature of approximately 200 °C.

Supplementary Notes

Supplementary Note 1. Energy and area balance analysis of utilizing solar heating and radiative cooling for CO_2 capture–release cycles

In this analysis, we assume the use of a Janus-type prototype device, though in practice the system may be implemented using a variety of specific configurations. We assume the energy input to our device during sunlight heating, or the energy output during radiative cooling are positive terms. To simplify the analysis, we hypothesize that there is no temperature gradient between the sunlight absorber and radiative cooling material; namely, their temperatures are the same. The thermal capacity of the device, except for zeolite absorbent and gas uptake, is not counted.

During the solar heating process, the sunlight absorber faces up, while the radiative cooling material faces down. The energy transfer process is described as follows:

$$Q_{\mathrm{H}} = \int_0^{t_{\mathrm{H}}} \begin{pmatrix} A_{\mathrm{s}} \left(P_{\mathrm{sun}} - (P_{\mathrm{emi,S}}(T_{\mathrm{H}}) - P_{\mathrm{atm}}(T_{\mathrm{amb}})) - P_{\mathrm{con,S}}(T_{\mathrm{H}}, T_{\mathrm{amb}}) \right) \\ -A_{\mathrm{RC}} \left(\left((P_{\mathrm{emi,RC}}(T_{\mathrm{H}}) - P_{\mathrm{grou}}(T_{\mathrm{grou}}) \right) + P_{\mathrm{con,RC}}(T_{\mathrm{H}}, T_{\mathrm{amb}}) \right) \end{pmatrix} dt \tag{S1}$$

where Q_{H} is the heat energy obtained via solar heating, t_{H} is the duration of solar heating in each desorption-adsorption cycle, and P_{sun} is the incident sunlight intensity. A_{s} and A_{RC} are the surface areas of the sunlight absorber and radiative cooling material, respectively. $P_{\mathrm{emi,S}}(T_{\mathrm{H}}) - P_{\mathrm{atm}}(T_{\mathrm{amb}})$ and $P_{\mathrm{con,S}}(T_{\mathrm{H}}, T_{\mathrm{amb}})$ are the radiative and conductive-convective thermal losses to the ambient via the upper sunlight absorber at heating mode, respectively. $P_{\mathrm{emi,RC}}(T_{\mathrm{H}}) - P_{\mathrm{grou}}(T_{\mathrm{grou}})$ and $P_{\mathrm{con,RC}}(T_{\mathrm{H}}, T_{\mathrm{amb}})$ are the radiative and conductive-convective thermal losses via the bottom radiative cooling material, respectively. T_{H}, T_{amb}, and T_{grou} are the temperatures of the sunlight absorber at heating mode, ambient, and ground, respectively.

Further,

$$P_{\mathrm{sun}} = \cos\theta_{\mathrm{sun}} \int_0^{\infty} d\lambda I_{\mathrm{sun}}(\lambda) \alpha_{\mathrm{S}}(\lambda, \theta_{\mathrm{sun}}) \tag{S2}$$

$$P_{\text{emi,S}}(T_{\text{H}}) - P_{\text{atm}}(T_{\text{amb}}) = \int d\Omega \cos\theta \int_0^{\infty} d\lambda I_{\text{BB}}(T_{\text{H}}, \lambda)\varepsilon_{\text{S}}(\lambda, \theta) - \int d\Omega \cos\theta \int_0^{\infty} d\lambda I_{\text{BB}}(T_{\text{amb}}, \lambda)\varepsilon_{\text{atm}}(\lambda, \theta)\alpha_{\text{S}}(\lambda, \theta) \quad \text{(S3)}$$

$$P_{\text{emi,RC}}(T_{\text{H}}) - P_{\text{grou}}(T_{\text{grou}}) = \int d\Omega \cos\theta \int_0^{\infty} d\lambda I_{\text{BB}}(T_{\text{H}}, \lambda)\varepsilon_{\text{RC}}(\lambda, \theta) - \int d\Omega \cos\theta \int_0^{\infty} d\lambda I_{\text{BB}}(T_{\text{grou}}, \lambda)\varepsilon_{\text{grou}}(\lambda, \theta)\alpha_{\text{RC}}(\lambda, \theta) \quad \text{(S4)}$$

$$P_{con,\text{S}}(T_{\text{H}}, T_{\text{amb}}) = h_{\text{s}}(T_{\text{H}} - T_{\text{amb}}) \quad \text{(S5)}$$

$$P_{con,\text{RC}}(T_{\text{H}}, T_{\text{amb}}) = h_{\text{RC}}(T_{\text{H}} - T_{\text{amb}}) \quad \text{(S6)}$$

in which, θ_{sun} is the direction of the incoming sunlight, λ is wavelength, I_{sun} is the spectral radiance of the sun, $\alpha_{\text{S}}(\lambda, \theta_{\text{sun}})$ is the absorptivity of sunlight absorber. Ω is a solid angle and θ is the angle between the direction of the solid angle and the normal direction of the surface. I_{BB} is the spectral radiance of a blackbody (temperature and wavelength dependent). $\varepsilon_{\text{S}}(\lambda, \theta)$, $\varepsilon_{\text{atm}}(\lambda, \theta)$, $\varepsilon_{\text{RC}}(\lambda, \theta)$ and $\varepsilon_{\text{grou}}(\lambda, \theta)$ are the emissivity of the sunlight absorber, ambient, radiative cooling material, and ground at a wavelength λ and angle θ. $\alpha_{\text{S}}(\lambda, \theta)$ and $\alpha_{\text{RC}}(\lambda, \theta)$ are the absorptivity of the sunlight absorber and radiative cooling material, respectively. *h* is a combined non-radiative heat transfer coefficient that includes conductive and convective heat exchanges. The subscripts of "s" and "RC" denote the sunlight heating side and radiative cooling side, respectively.

Based on the initial assumptions, all the energy harvested from sunlight heating and radiative cooling is used for changing the temperature of the zeolite absorbent and absorbed carbon dioxide.

$$Q_{\text{H}} = m_{\text{z}} C_{\text{p,z}} \Delta T_{\text{Des}} + \left(\gamma(T_{\text{c}}) m_{\text{z}} + \kappa\right)\left(C_{\text{p,CO2}} \Delta T_{\text{Des}} + H_{\text{ads}}\right) \quad \text{(S7)}$$

where, m_{z} is the mass of zeolite adsorbent. $C_{\text{p,z}}$ and $C_{\text{p,CO2}}$ are the thermal capacities of zeolite and carbon dioxide, respectively. ΔT_{Des} is the temperature changes during desorption a process. $\gamma(T_{\text{c}})$ and κ are the cooling temperature-dependent carbon dioxide uptake coefficients of the zeolite. H_{ads} denotes the adsorption heat (per unit weight) of the zeolite adsorbent for carbon dioxide.

In the radiative cooling process, the radiative cooling material is flipped up with the sunlight absorber faces down. The energy transfer process is as follows:

$$Q_c = \int_0^{t_C} \begin{pmatrix} A_{RC}\left(-P_{sun} + \left(P_{emi,RC}(T_C) - P_{atm}(T_{amb})\right) - P_{con,RC}(T_C, T_{amb})\right) \\ +A_S\left(\left(P_{emi,S}(T_C) - P_{grou}(T_{grou})\right) - P_{con,S}(T_C, T_{amb})\right) \end{pmatrix} dt \tag{S8}$$

where Q_C is the cold energy obtained via radiative cooling and t_C is the duration of radiative cooling in each desorption-adsorption cycle. T_C denotes the temperature of a radiative cooling material in cooling mode.

The above terms can be further expressed as,

$$\begin{aligned}(P_{emi,RC}(T_C) - P_{atm}(T_{atm}) &= \int d\Omega \cos\theta \int_0^\infty d\lambda I_{BB}(T_C, \lambda)\varepsilon_{RC}(\lambda, \theta) \\ &-\int d\Omega \cos\theta \int_0^\infty d\lambda I_{BB}(T_{atm}, \lambda)\varepsilon_{atm}(\lambda, \theta)\alpha_{RC}(\lambda, \theta)\end{aligned} \tag{S9}$$

$$\begin{aligned}P_{emi,S}(T_C) - P_{grou}(T_{grou}) &= \int d\Omega \cos\theta \int_0^\infty d\lambda I_{BB}(T_H, \lambda)\varepsilon_S(\lambda, \theta) \\ &-\int d\Omega \cos\theta \int_0^\infty d\lambda I_{BB}(T_{grou}, \lambda)\varepsilon_{grou}(\lambda, \theta)\alpha_S(\lambda, \theta)\end{aligned} \tag{S10}$$

$$P_{con,RC}(T_H, T_{amb}) = h_{RC}(T_{amb} - T_C) \tag{S11}$$

$$P_{con,S}(T_H, T_{amb}) = h_S(T_{amb} - T_C) \tag{S12}$$

We therefore have the energy balance of:

$$Q_c = m_z C_{p,z} \Delta T_{Ads} + \left(\gamma(T_c) m_z + \kappa\right)\left(C_{p,CO2} \Delta T_{Ads} + H_{ads}\right) \tag{S13}$$

in which, ΔT_{Ads} is the temperature change during the adsorption process driven by radiative cooling.

The above energy and area balance analysis incorporates impacts from local weather and geography conditions by P_{sun}, T_{amb}, T_{grou}, $\varepsilon_{atm}(\lambda, \theta)$, $\varepsilon_{grou}(\lambda, \theta)$, h_S, and h_{RC}. These data can be extracted from local history records. By solving equations of S1, S7, S8, and S13 together with some presuppositions, we can then obtain the required surface areas of the sunlight absorber and radiative cooling materials. Presuppositions include t_H, t_C, T_H, T_C,

m_z, ΔT_{Des}, and ΔT_{Ads}. The deviation due to the idealized assumptions for simplifying analysis can be compensated by relatively increasing the areas of sunlight absorbers and radiative cooling materials in practice (*e.g.,* multiplying a factor, a common method used in engineering).

Supplementary Note 2. Techno-economic analysis

The primary analysis only considers the initial investment for material synthesis and operational process during the adsorption–desorption cycles. The synthesis cost includes chemical consumption and energy usage, while the operational cost mainly refers to the energy required for adsorbent regeneration.

A. Material Synthesis Cost

During synthesis, the major chemical costs arise from kaolin, sodium hydroxide, and calcium chloride (used here as a substitute for calcium nitrite as the Ca^{2+} source), with assumed market prices of $50, $150, and $170 per ton, respectively, based on publicly available data. The cost of water is neglected. Unless otherwise noted, chemical consumption and temperature conditions follow those described in the Methods section.

Energy consumption during synthesis comes from the calcination, hydrothermal reaction, and ion-exchange steps. For simplicity, only the heating energy is considered, and contributions from mixing, separation, and drying are omitted. All heating energy is assumed to come from electricity with a 95% conversion efficiency at a price of $0.08 per kWh, and heat-recovery systems are assumed to recycle 90% of the thermal energy.

1) Calcination energy consumption

$$E_1 = m_\mathrm{k} C_{\mathrm{p,k}} \Delta T_1 \tag{S14}$$

where m_k is the mass usage of kaolin, $C_{\mathrm{p,k}}$ is the specific thermal capacity of kaolin and is assumed to be 1.1 kJ kg^{-1} K^{-1}, ΔT_1 is the temperature change during the calcination process.

2) Hydrothermal reaction

$$E_2 = m_\mathrm{k}' C_{\mathrm{p,k}} \Delta T_2 + m_\mathrm{S} C_{\mathrm{p,S}} \Delta T_2 + m_{\mathrm{W},2} \Delta H_2 \tag{S15}$$

in which, m_k' is the mass of kaolin after calcination. The mass loss during the calcination process in comparison to m_k is ~6.5%, which is determined via the TGA test result (Fig. S10). The m_S and $m_{W,2}$ are the masses of sodium hydroxide and water used in this reaction are, respectively. For m_S, we only consider the contribution from zeolite formation, which accounts for approximately 17% of the obtained sample, as determined by the XRF analysis (Fig. S17). The $C_{p,S}$ and ΔH_2 are the specific thermal capacity of sodium hydroxide and enthalpy change of water, respectively, which are assumed to be 1.5 kJ kg^{-1} K^{-1} and 284.7 kJ kg^{-1}. The ΔT_2 is the temperature change during the hydrothermal process.

3) **Ion exchange**

$$E_3 = m_Z C_{p,Z} \Delta T_3 + m_C C_{p,C} \Delta T_3 + m_{W,3} \Delta H_3 \quad \text{(S16)}$$

where m_Z, m_C, and $m_{W,3}$ denote the masses of zeolite material, calcium chloride, and water used in this ion exchange process, respectively. The m_Z increases by ~20% in comparison to m_k'. The consumption of calcium chloride was determined via the result of element content obtained via XRF test (~9.5%). The $C_{p,Z}$, $C_{p,C}$, and ΔH_3 are the specific thermal capacity of zeolite, the specific thermal capacity of calcium chloride, and the enthalpy change of water, respectively. They are assumed to be 0.92 kJ kg^{-1} K^{-1}, 0.66 kJ kg^{-1} K^{-1}, and 251.1 kJ kg^{-1}, respectively. The ΔT_3 denotes the temperature change during the ion exchange process. According to our experimental results, we assume ~20% of the sample is lost during the ion exchange process.

B. Operational Cost during CO_2 Capture Cycles

The dominant operational cost arises from adsorbent regeneration by heating. We here consider the regeneration method used in the TGA cycling test.

$$E_D = \Big((m_{\mathrm{CZ}} C_{\mathrm{p,CZ}} \Delta T_D + m_{\mathrm{CO_2}} C_{\mathrm{p,CO_2}} \Delta T_D) \times 19/20 + (m_{\mathrm{CZ}} C_{\mathrm{p,CZ}} \Delta T_D' + m_{\mathrm{CO_2}} C_{\mathrm{p,CO_2}} \Delta T_D') \times 1/20 \Big) + m_{\mathrm{CO_2}} E / 44 \tag{S17}$$

in which, m_{CZ} and $m_{\mathrm{CO_2}}$ are the masses of zeolite after Ca^{2+} ion exchange and the adsorbed carbon dioxide, respectively. The m_{CZ} is nearly the same as m_{k}'. The $C_{\mathrm{p,CZ}}$ and $C_{\mathrm{p,CO_2}}$ are the specific thermal capacities of zeolite and carbon dioxide, which are assumed to be 0.92 kJ kg^{-1} K^{-1} and 0.85 kJ kg^{-1} K^{-1}, respectively. The ΔT_D and $\Delta T_D'$ are the temperature increases during the regeneration process from room temperature, with ΔT_D denoting the low temperature regeneration and $\Delta T_D'$ representing the high temperature regeneration, respectively. The low regeneration temperature is 150 °C, while the high regeneration temperature is 450 °C, and the average regeneration efficiency is approximately 85%. We assume that the zeolite requires high-temperature regeneration after every 19 low-temperature regeneration cycles. E is the desorption heat of carbon dioxide, and is assumed as 50 J $mmol^{-1}$. We assume the heat energy for regeneration is provided by sunlight at a price of $0.03 per kWh by referring to previous literatures [1, 2].

The price of harvesting radiative cooling energy is not found in previous literature. Thus, we here will do an estimation based on a selective radiative cooling material, es-PEO film for example [3]. According to the method and results reported in the previous work, the main cost includes raw material and the electrospinning process. The materials include 25 μm PEO, 10 μm Aluminum film, and Acetonitrile (nine times the weight of PEO). The prices are assumed $6/kg, $2.8/kg, and $14/kg, respectively, based on online data. The electrospinning process includes energy consumption from generating high voltage and pump, which are presumed as $8.5*10^{-2}$ kWh h^{-1} and $2.5*10^{-2}$ kWh h^{-1}, respectively [4]. We assume 10 hours of electrospinning is used for preparing the es-PEO. The price of such a radiative cooling material is $3.9 m^{-2}. We refer to the reported relationship between

radiative cooling power and temperature of a selective radiative cooling material to predict the cooling performance. When the radiative cooling material is at 0 °C, the cooling power is around 50 W m^{-2}. We assume the radiative cooling material works 6 hours every day and has a life time of 2 years (300 days every year). The unit cost is estimated to \$0.02 kWh^{-1}, which is very close to the unit cost of utilizing sunlight heating of \$0.03 per kWh. As the heating and cooling process are converse processes, we hypothesize that the final operational cost is twice as much as the price due to sunlight heating-based regeneration to simplify the estimation.

C. Final Cost Estimation

The total cost is obtained by summing the material synthesis and operational costs, normalized to US \$/ton of captured CO_2.

Here is an example. Based on the adsorption isotherms at 0 °C, we extrapolate the carbon dioxide uptake is ~4.27 mmol g^{-1} sample under15% carbon dioxide. The results in Fig. S11 suggest that the cost for capturing per ton of carbon dioxide decreases with cycling number. When the cycling number exceeds 50, the cost will be lower than the convection technologies used in three major sectors [5]. Our analysis also finds that raw material costs dominate the total expenditure, especially at low cycling numbers. Future large-scale production is expected to further reduce these material costs.

This first-pass estimation does not fully consider infrastructure, labor, transportation, and maintenance costs. Subsequent studies should incorporate detailed process simulations for more accurate cost assessments.

Supplementary Note 3. Energy consumption analysis of solar heating and radiative cooling powered CO_2 capture–release cycles

We here consider the regeneration method used in the TGA cycling test. The regeneration energy consumption is estimated via Eq. S17. Here, we assume that radiative cooling materials can cool the sample temperature from ambient temperature (20 °C) to 0 °C to simplify the analysis. Based on the adsorption isotherms at 0 °C, we extrapolate that the carbon dioxide uptake is ~4.27 mmol g^{-1} sample under 15% carbon dioxide. Besides, we suppose 90% of the heat energy will be recovered and reused. As a result, the energy consumption for releasing 1 ton of CO_2 from the zeolite adsorbent is ~60 kWh of heat. While for the conventional temperature swing adsorption systems, the minimum required energy is ~500 kWh heat per ton CO_2 [6-8].

Radiative cooling enhances CO_2 uptake during the adsorption step. In conventional industrial processes, active cooling is seldom used because the required electricity typically outweighs the gain in adsorption capacity. Radiative cooling overcomes this limitation by providing a fully passive cooling pathway. Although a direct energy-consumption comparison for cooling-enhanced CO_2 capture is not possible due to the absence of industrial analogues, it is essential to demonstrate through techno-economic and carbon-footprint analysis that radiative cooling functions as a cooling technology with good cost-effectiveness and net-negative carbon emissions.

In the above analysis, we obtain the price of such a radiative cooling material is \$3.9 m^{-2}. To evaluate the cooling benefit, we refer to the reported relationship between temperature and cooling power for selective radiative coolers [9]. At 0 °C, the cooling power is approximately 50 W m^{-2}. Assuming 6 hours of operation per day and a lifetime of 2 years (300 days per year), the cumulative cooling energy harvested reaches ~180 kWh m^{-2}. If the cooling energy provide by 1 m^2 of radiative cooling material were otherwise supplied by electricity, the energy cost is 60 kWh, assuming a representative cooling COP of 3 [10]. If an electricity price is \$0.08 per kWh, this equals to \$4.8, exceeding the material fabrication cost.

We further estimate the carbon footprint of the es-PEO film following the procedure reported in [3]. The carbon intensities of PEO, aluminum film, and acetonitrile are taken as 3.5, 18.2, and 5.6 kg CO_2-Eq per kg, respectively (the latter two from the ecoQuery database). In the absence of direct data for PEO, its value is approximated using the average carbon intensity of plastics reported in [11]. The energy consumption of electrospinning comes mainly from the high-voltage supply and the feeding pump, and the carbon intensity of electricity is taken as 0.45 kg CO_2-Eq per kWh. Overall, the CO_2 footprint for preparing one square meter of es-PEO film is estimated to be 2.5 kg CO_2-Eq.

If this cooling energy were otherwise provided by electricity, its carbon footprint would be ~0.16 kg CO_2-Eq per kWh of cooling energy, assuming a representative cooling COP of 3 [10]. Under this assumption, the avoided emissions amount reaches ~28.8 kg CO_2-Eq m^{-2}. Subtracting the 2.5 kg CO_2-Eq m^{-2} required for fabrication yields a net footprint of approximately –26.3 kg CO_2-Eq m^{-2}, indicating that radiative cooling represents a carbon-negative pathway.

Supplementary Note 4. Carbon footprint estimation

In this estimation, carbon adsorption enabled by zeolite adsorbent contribute negative carbon footprint, while the carbon emission mainly comes from adsorbent synthesis and operation. According to the synthesis method, the raw materials include kaolin clay, sodium hydroxide, and calcium chloride. Based on the data from ecoQuery database, their carbon footprint are 0.214, 0.778, and 0.473 kg CO_2-Eq per kg, respectively. The energy consumption of electricity has a carbon intensity of 0.45 kg-Eq per kWh. As a result, the carbon footprint for producing the zeolite adsorbent is 0.61 kg CO_2-Eq per kg.

During the operation, the carbon footprint of solar heating is 24 g CO_2-Eq kWh^{-1} [12, 13]. The above estimations in Supplementary Note 3 shows that the carbon emission when preparing the es-PEO film is 2.5 kg CO_2-Eq per m^2. Given the cooling energy harvesting during the 2 years of lifetime, the carbon intensity of utilizing radiative cooling is 14 g CO_2-Eq per kWh, which is very close to the value of sunlight heating. Therefore, we duplicate the carbon footprint of solar heating to estimate the carbon emission during operation to simplify the calculations.

Finally, we summarize the carbon capture and carbon emissions and plot the carbon footprint as a function of the adsorption–desorption cycling number. We find that the carbon footprint becomes negative after 4 cycles, indicating that the initial emissions associated with adsorbent synthesis are fully offset by its carbon capture capacity. With further cycling, the zeolite adsorbent enables substantial net carbon removal as its cumulative capture continues to increase.

Supplementary Note 5. Material and water balances in alkaline treatment and Ca-exchange

Based on the preparation method and XRF result, we obtain the material and water balances (Figs. S17 and S18). As only a small amount of zeolite is lost during the synthesis process, the water consumption is negligible. It is also found that 42% of sodium hydroxide and 96% of sodium hydroxide and calcium nitrate can be reused. Separating the zeolite from the solution, in fact, is quite easy. Natural sedimentation can separate most solids from the solution (Fig. S19). Filtration or centrifugation can further facilitate. After the solid and liquid separation, the solutions with water, sodium hydroxide, and calcium hydroxide can be reused by regularly adding new chemicals to each reaction batch. The reagent recovery will benefit for lowering the material synthesis cost.

Supplementary Note 6. Tolerance to kaolin impurities in the alkaline treatment and Ca-exchange steps

We evaluated the tolerance of our synthesis process to the intrinsic impurities in the raw kaolin. XRF analysis indicates that the main impurities with concentrations above 1000 ppm are Mg, K, Ti, and Fe, appearing only at the ppm level (1054–8841 ppm, equivalent to approximately 0.1–0.8 wt%). During the alkaline treatment and Ca-exchange steps, the slurry is prepared by adding water at five times and fifty times the solid mass, respectively, which further dilutes these impurity cations by additional orders of magnitude. After dispersion in the reaction slurry, their effective concentrations become extremely low. In contrast, the active reagents, 3 M NaOH in the alkaline treatment and 1 M Ca^{2+} in the ion-exchange step, are present at much higher concentrations and therefore dominate the chemical environment. As a result, the ppm-level impurities do not compete with Na^{+} or Ca^{2+} and do not participate in dissolution, framework restructuring, or ion-exchange reactions. Accordingly, the synthesis process exhibits strong tolerance to typical kaolin impurities.

Supplementary Figures

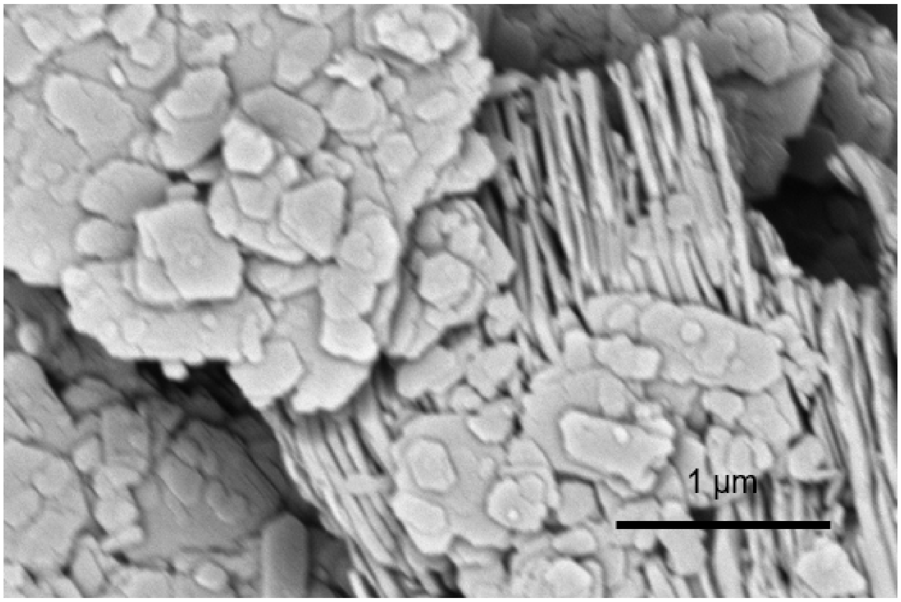

Supplementary Fig. 1. A SEM image shows the lamellar structure of kaolin clay.

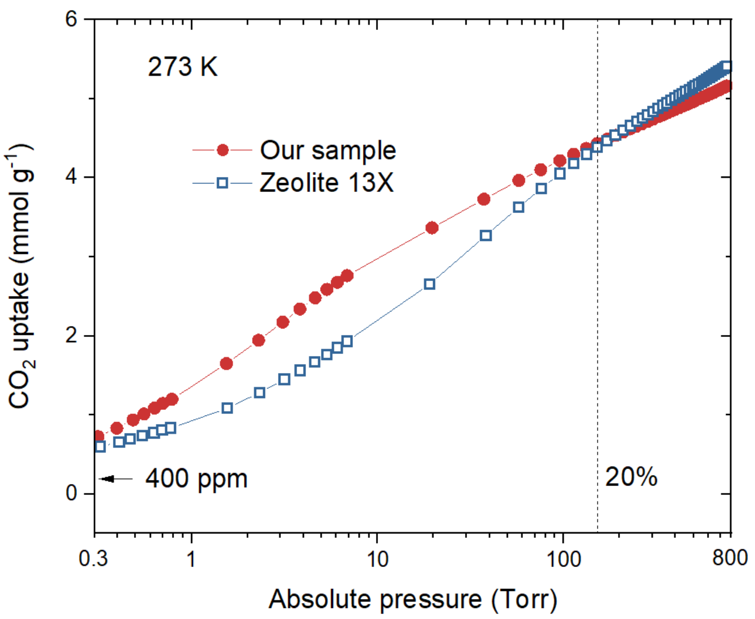

Supplementary Fig. 2. Carbon dioxide isotherms of our sample and commercialized zeolite 13X. Our sample shows better performance than the commercial product over a wide carbon dioxide pressure range, which covers the main industrial scenarios of carbon capture, from direct air capture, to natural gas sweetening, flue gas capture, and hydrogen production.

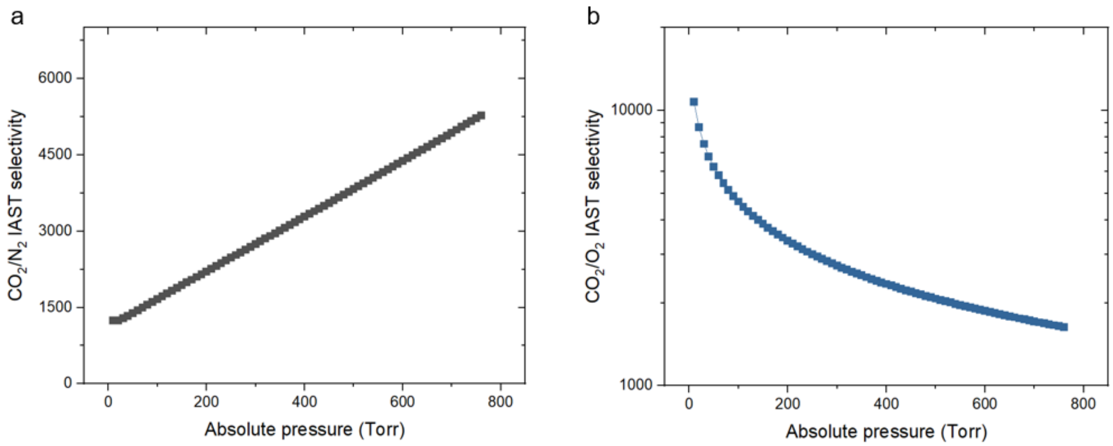

Supplementary Fig. 3. Adsorption selectivity of the as-prepared LTA zeolite at 288 K. (a) For CO_2/N_2. (b) For CO_2/O_2. The calculations were completed via the IAST++ software based on the Ideal Adsorbed Solution Theory (IAST). Calculations are performed assuming a CO_2 feed composition of 15%.

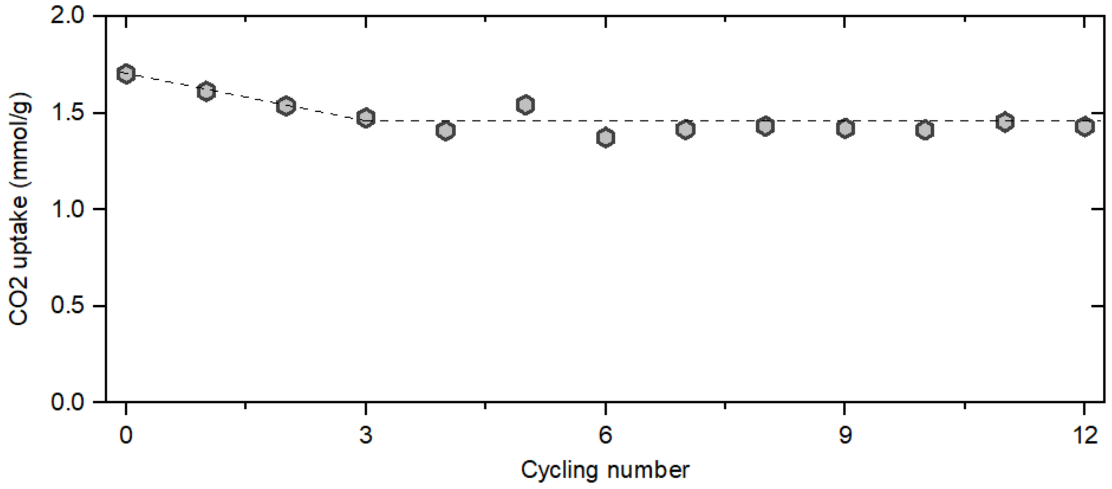

Supplementary Fig. 4. Cycling tests by temperature swing. The sample was first activated at 300 °C for 3 hours. Then, repeated adsorption (at 20 °C) and desorption (at 160 °C, 3 hours) cycles were down via breakthrough setups.

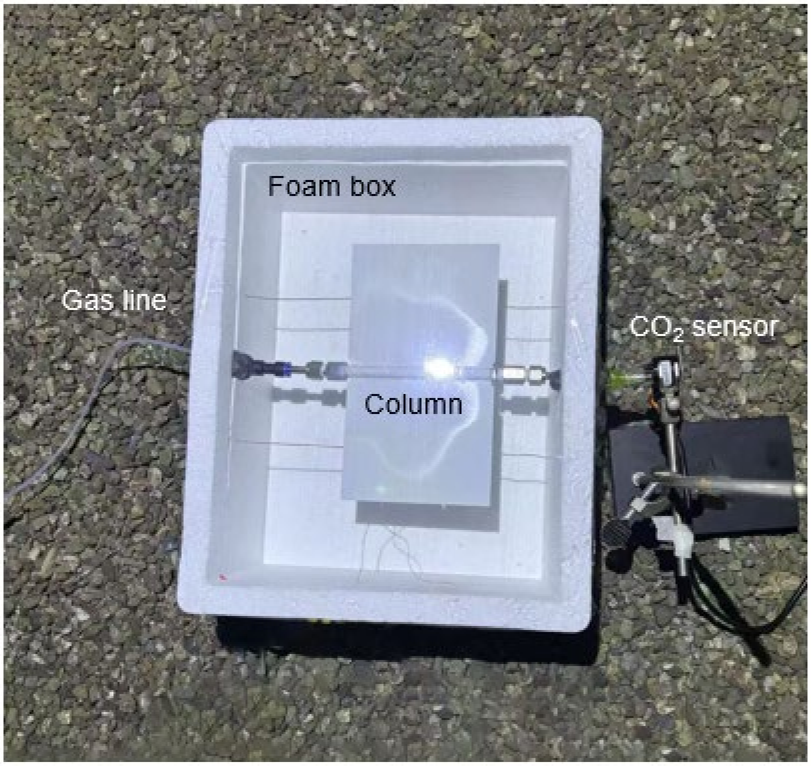

Supplementary Fig. 5. A photograph of the experiment setups for enabling adsorption enhancement by utilizing radiative cooling.

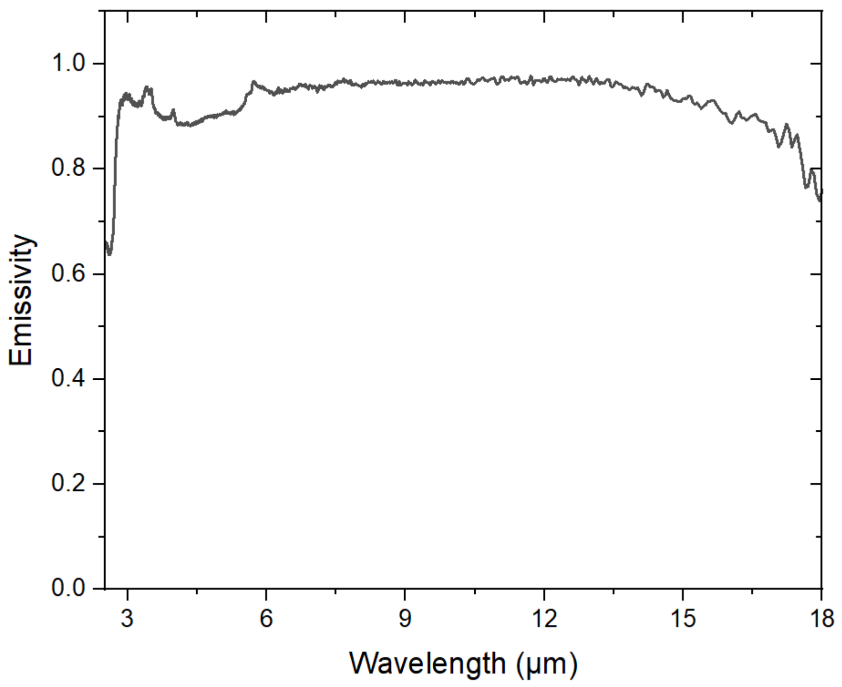

Supplementary Fig. 6. Mid-infrared emissivity spectrum of the radiative cooling material used in this study.

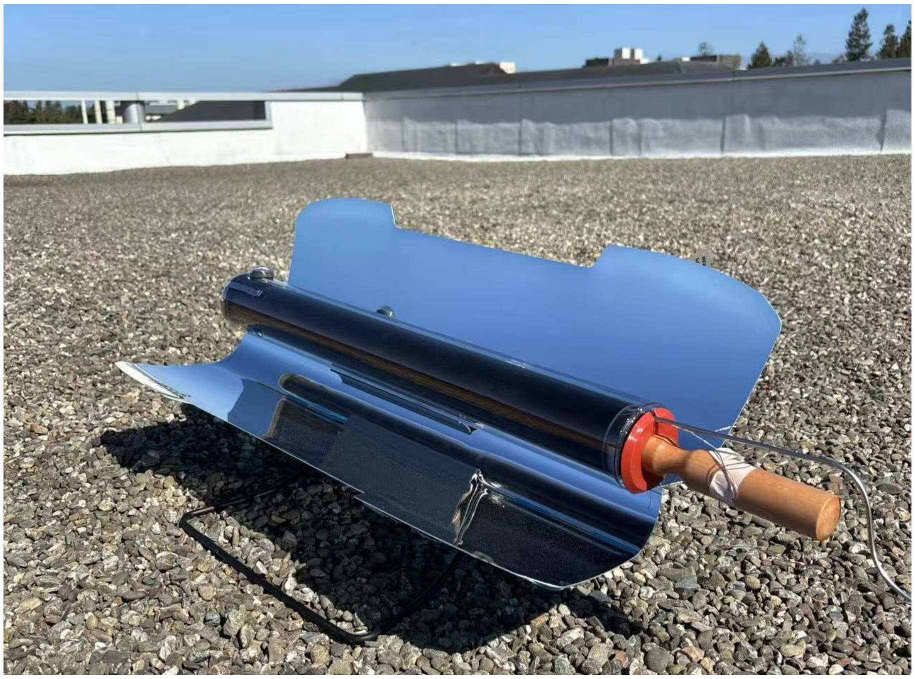

Supplementary Fig. 7. A photograph shows solar heating via an evacuated solar heating tube. An adsorption column with LTA zeolite is mounted inside the tube for regeneration.

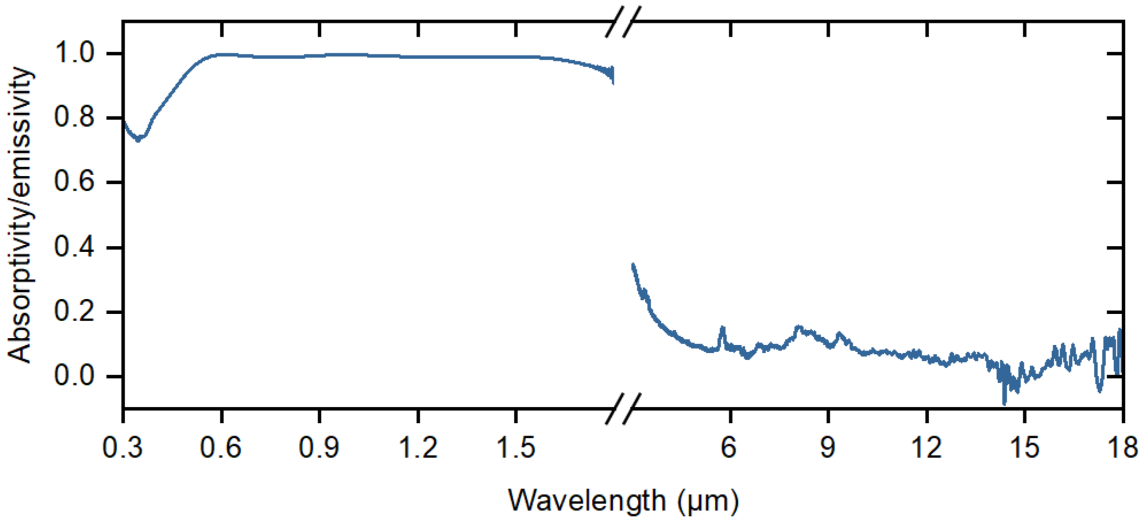

Supplementary Fig. 8. Optical spectrum of the solar heating material used in this study.

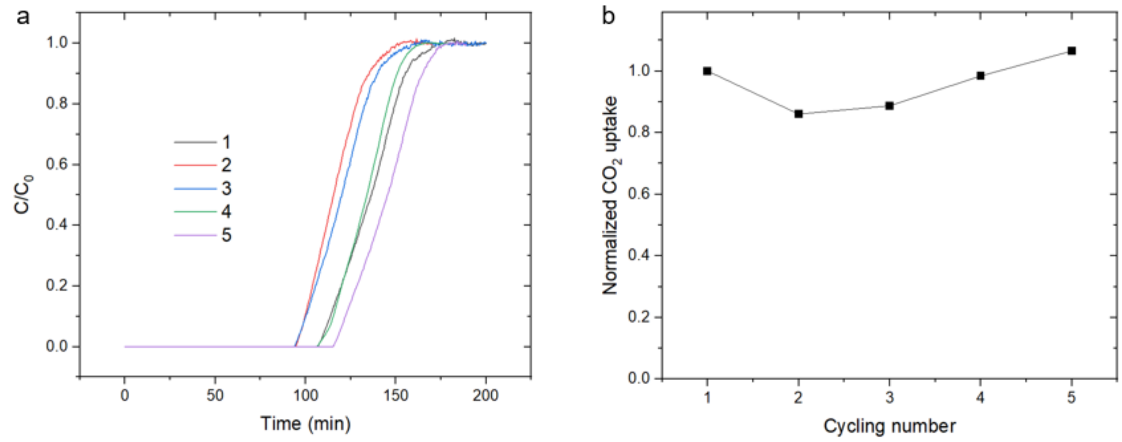

Supplementary Fig. 9. Breakthrough results over multiple cycles, achieved by integrating solar heating for adsorbent regeneration and radiative cooling to enhance adsorption. (a) Breakthrough results. (b) Normalized CO_2 uptake by the first cycle. Variations in the data are attributed to weather-dependent conditions during testing.

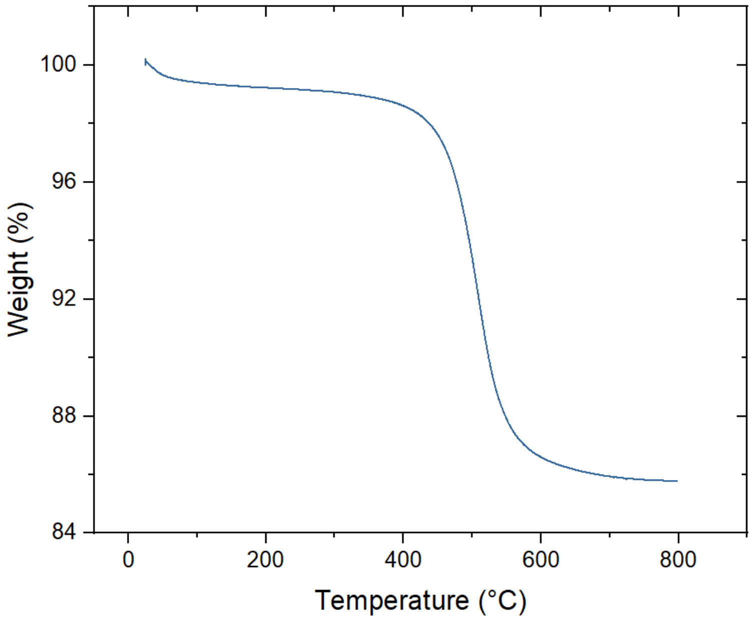

Supplementary Fig. 10. TGA result of kaolin clay.

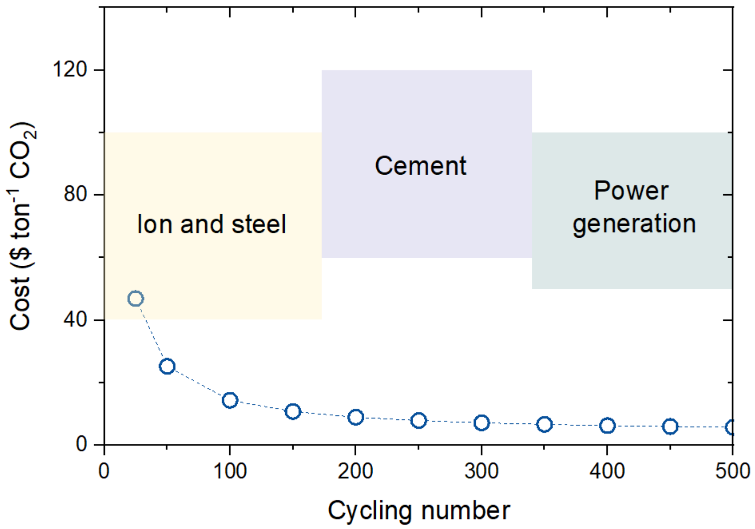

Supplementary Fig. 11. A preliminary cost analysis shows that increasing the number of adsorption–desorption cycles substantially lowers the carbon-capture cost, offering potential economic advantages over conventional technologies across three major sectors [5].

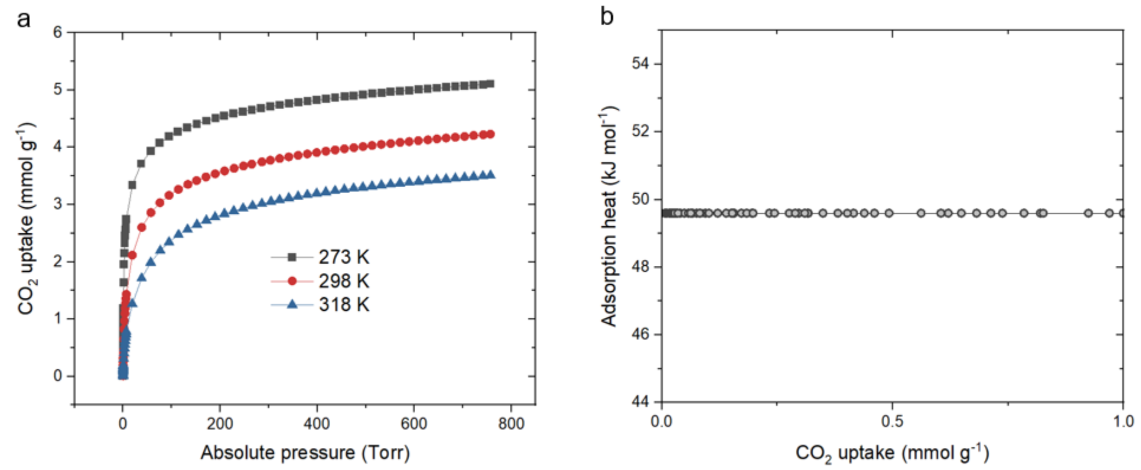

Supplementary Fig. 12. Adsorption heat of the synthesized zeolite. (a) Adsorption isotherms of as-prepared zeolite at 273 K, 298 K, and 318 K. (b) Adsorption heat at low surface coverage calculated based on the results in a) via AIM software.

At low coverage, the adsorption heat of approximately ~50 kJ mol^{-1} is lower than that of amine-based adsorbents, typically 60–100 kJ mol^{-1} [14]. With increasing CO_2 loading, the adsorption heat is expected to decrease, which can be attributed to the progressive occupation of energetically weaker adsorption sites and the increasing contribution from micropore filling.

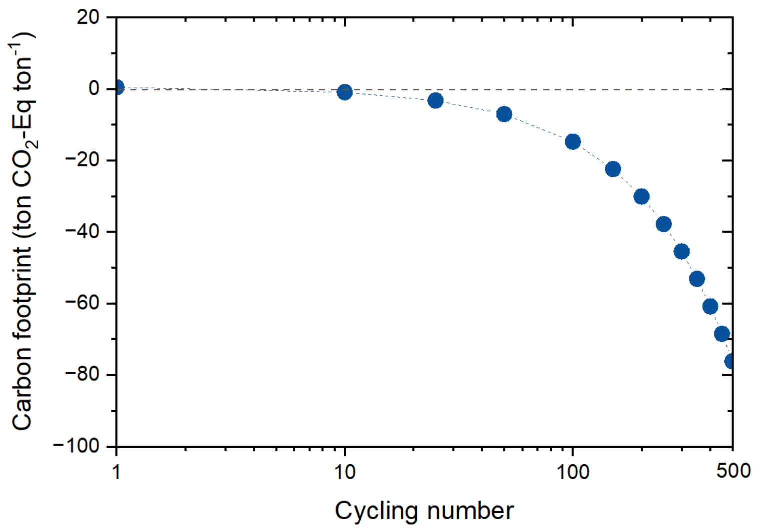

Supplementary Fig. 13. Estimated carbon footprint. The carbon capture pathway reported in this work is capable of negative emissions after 4 adsorption-desorption cycles.

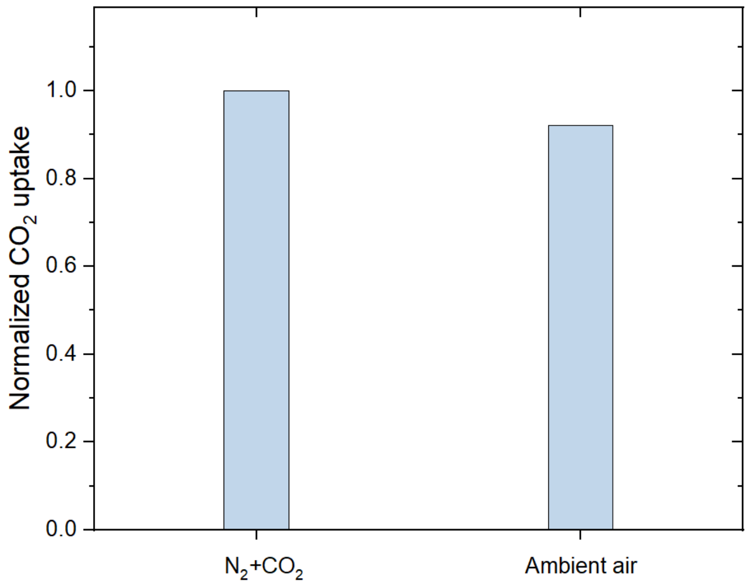

Supplementary Fig. 14. Impacts of competitive adsorption.

In both tests, the samples were activated at 250 °C under He flow. The CO_2 concentrations were controlled to be comparable and were further calibrated to an equivalent basis by assuming a linear relationship between gas uptake and CO_2 concentration.

The competitive adsorption of H_2O, NO_x, and SO_x decreases the CO_2 uptake of the as-prepared zeolite by about 8%. Among these species, H_2O is the primary contributor and is a widely recognized factor in zeolite-based CO_2 adsorption. Future work may explore mitigation approaches such as hydrophobic surface modification or system-level moisture management, including the use of an upstream dehumidification step [15].

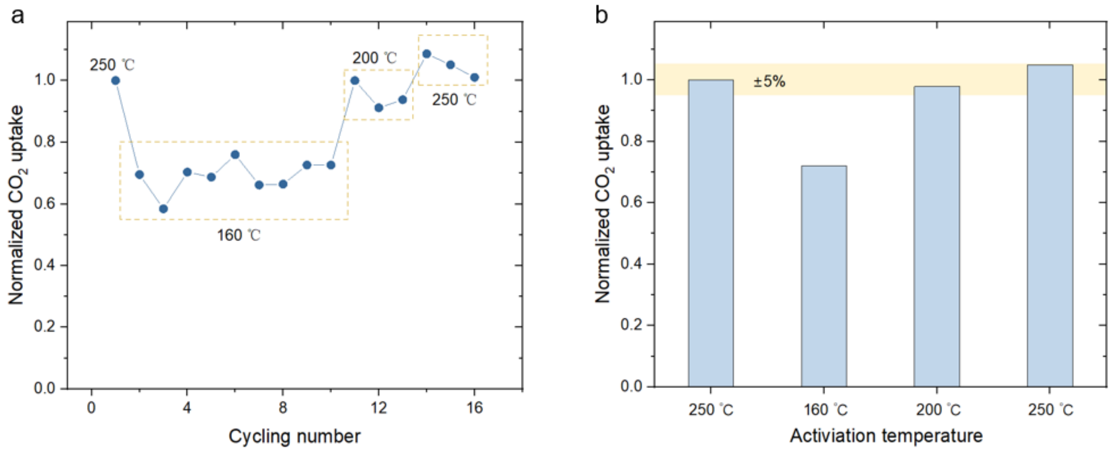

Supplementary Fig. 15. Cycling test under direct air capture conditions. (a) CO_2 uptake in each cycle. The sample was initially activated at 250 °C, followed by regeneration at 160 °C (9 cycles), 200 °C (3 cycles), and 250 °C (3 cycles) under He flow. The CO_2 concentrations were kept comparable across cycles and further normalized to an equivalent basis by assuming a linear relationship between gas uptake and CO_2 concentration. (b) Mean CO_2 uptake values calculated from the data shown in (a).

A regeneration temperature of 160 °C enabled a ~72% capacity recovery. Increasing the regeneration temperature to 200–250 °C facilitated an almost complete restoration of the original CO_2 uptake (>95%).

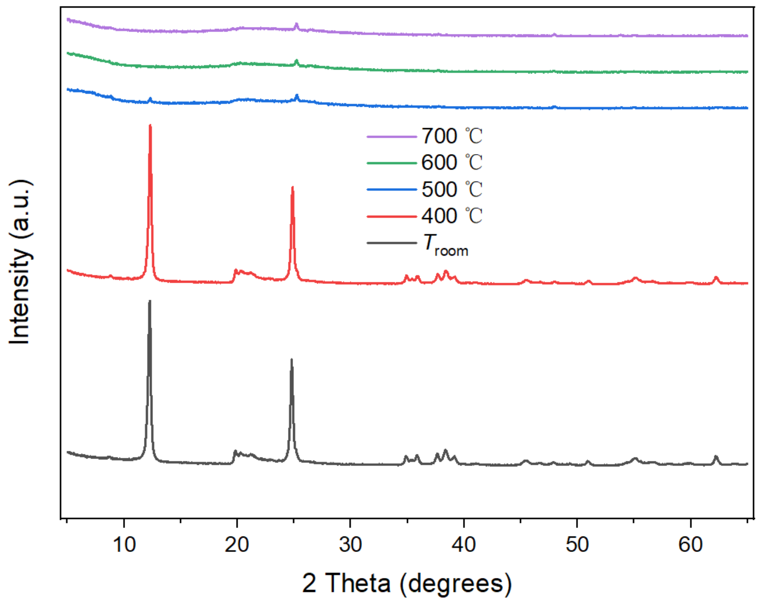

Supplementary Fig. 16. XRD patterns of kaolin calcinated at different temperatures. When the calcination temperature is higher than 500 °C, kaolin is turned into an amorphous state. This is important for increasing the reactivity of kaolin in the hydrothermal reactions to convert kaolin into zeolite. Therefore, we chose the lowest 500 °C as the calcination temperature to prepare the zeolite.

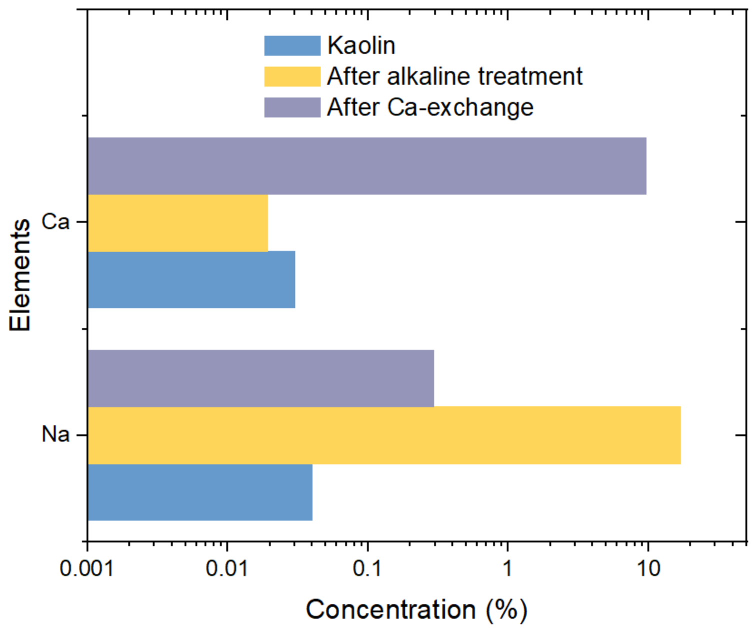

Supplementary Fig. 17. Concentration variations of the key metal elements during zeolite adsorbent synthesis.

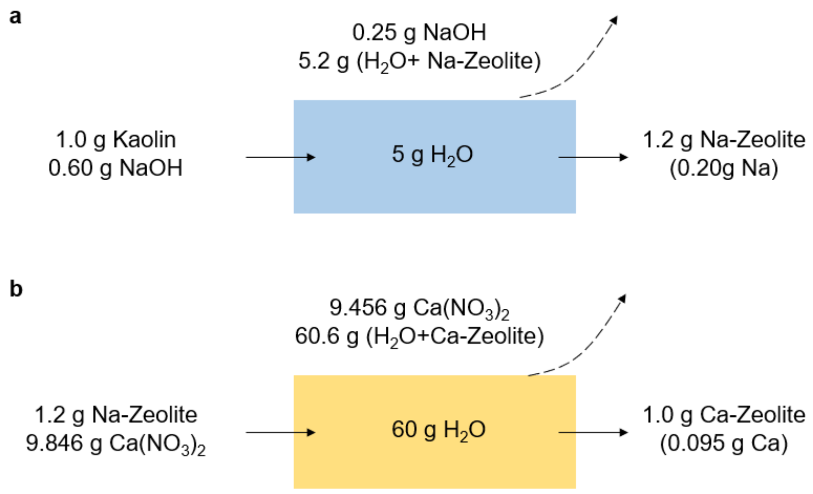

Supplementary Fig. 18. Material and water balance during zeolite adsorbent synthesis. Left is input, right is output, and top is the material/water that can be recycled and reused. The alkaline treatment (a) and Ca-exchange steps (b). Most water, 42% of sodium hydroxide, and 96% of calcium nitrate can be reused for the next batch of material synthesis after solid and liquid separation.

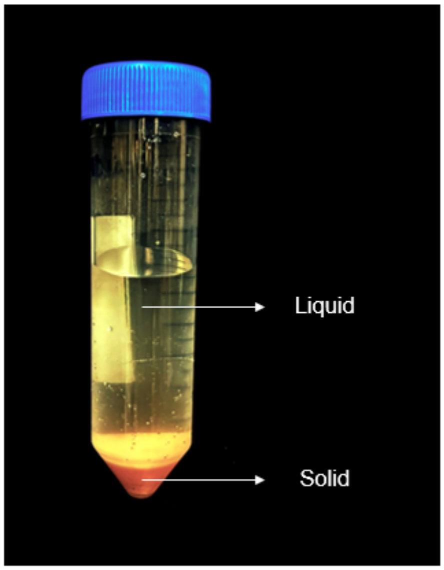

Supplementary Fig. 19. During the zeolite adsorbent synthesis process, solid and liquid separation can be easily realized by just natural sedimentation.

Supplementary References

1. Silviano-Mendoza HH, Martinez-Rodriguez G, Fuentes-Silva AL *et al.* Life cycle cost analysis of a Low-Temperature solar thermal system which delivers thermal and electric energy to a cotton dyeing industrial process. *Chem Eng Trans* 2023; **103**: 667-672.
2. Gilbert T, Menon AK, Dames C *et al.* Heat source and application-dependent levelized cost of decarbonized heat. *Joule* 2023; **7**: 128-149.
3. Li D, Liu X, Li W *et al.* Scalable and hierarchically designed polymer film as a selective thermal emitter for high-performance all-day radiative cooling. *Nat Nanotechnol* 2021; **16**: 153-158.
4. Malara A. Environmental concerns on the use of the electrospinning technique for the production of polymeric micro/nanofibers. *Sci Rep* 2024; **14**: 8293.
5. International Energy Agency. *Is carbon capture too expensive?* https://www.iea.org/commentaries/is-carbon-capture-too-expensive (18 December 2025, date last accessed)
6. Ziobrowski Z, Rotkegel A. Comparison of CO2 separation efficiency from flue gases based on commonly used methods and materials. *Materials* 2022; **15**: 460.
7. Aghel B, Janati S, Wongwises S *et al.* Review on CO2 capture by blended amine solutions. *Int J Greenhouse Gas Control* 2022; **119**: 103715.
8. Gao W, Liang S, Wang R *et al.* Industrial carbon dioxide capture and utilization: state of the art and future challenges. *Chem Soc Rev* 2020; **49**: 8584-8686.
9. Fan S, Li W. Photonics and thermodynamics concepts in radiative cooling. *Nat Photonics* 2022; **16**: 182-190.
10. Global Change Data Lab. *Air conditioning causes around 3% of greenhouse gas emissions. How will this change in the future?* https://archive.ourworldindata.org/20251125-173858/air-conditioning-causes-around-greenhouse-gas-emissions-will-change-future.html (18 December 2025, date last accessed)
11. Zheng J, Suh S. Strategies to reduce the global carbon footprint of plastics. *Nat Clim Chang* 2019; **9**: 374-378.

12. Milousi M, Souliotis M, Arampatzis G *et al.* Evaluating the environmental performance of solar energy systems through a combined life cycle assessment and cost analysis. *Sustainability* 2019; **11**: 2539.
13. Burkhardt III JJ, Heath GA, Turchi CS. Life cycle assessment of a parabolic trough concentrating solar power plant and the impacts of key design alternatives. *Environ Sci Technol* 2011; **45**: 2457-2464.
14. Wang Y, Qu L, Ding H *et al.* Distributed direct air capture of carbon dioxide by synergistic water harvesting. *Nat Commun* 2024; **15**: 9745.
15. Kolle JM, Fayaz M, Sayari A. Understanding the effect of water on CO2 adsorption. *Chem Rev* 2021; **121**: 7280-7345.
